# Crystallization of Poly(ε-caprolactone) in Poly(vinylidene fluoride)/Poly(ε-caprolactone) Blend

**DOI:** 10.3390/polym9020042

**Published:** 2017-01-28

**Authors:** Yang Kong, Yangmin Ma, Lele Lei, Xuechuan Wang, Haijun Wang

**Affiliations:** Shaanxi University of Science and Technology, Xi’an 710021, Shaanxi, China; kongyang@sust.edu.cn (Y.K.); mayangmin@sust.edu.cn (Y.M.); 1526875111@qq.com (L.L.); wangxc@sust.edu.cn (X.W.)

**Keywords:** poly(ε-caprolactone), poly(vinylidene fluoride), blend, crystallization

## Abstract

The crystallization behavior of poly(ε-caprolactone) (PCL) in a poly(vinylidene fluoride) (PVDF)/PCL blend as well as on a highly orientated PVDF substrate was studied by means of POM, DSC and TEM. The results show that the miscibility of the PVDF/PCL blend and the spherulitic morphology of PVDF varies with the blend ratio. For all the compositions, the pre-existing PVDF crystals accelerated the crystallization of PCL because the PVDF exhibits very strong nucleation ability toward PCL as reflected by the occurrence of heteroepitaxy and the transcrystallization of PBA on the PVDF substrate. This is associated with the perfect lattice matching between the PBA and PVDF crystals.

## 1. Introduction

As a simple and cost-effective way to improve the physical and mechanical properties of polymeric materials, polymer blending has attracted a broad interest [1,2,3,4]. Polymer blends can be miscible, partially miscible, or immiscible systems, including amorphous/amorphous, amorphous/crystalline and crystalline/crystalline components. As is known to all, the properties of all of these possible blends depend strongly on their morphologies and crystal structure. The crystallization behavior and related morphological control of polymer blends has consequently interested more and more theoreticians and experimentalists [5,6,7].

Comparatively speaking, the morphology of the crystalline/crystalline blend is more complicated than that of the other two systems, because the phase separation structure of this system is always changing during the sequential crystallization of the two components. It should be noted that the crystallization kinetics and morphology of crystalline/crystalline blends are far away from being really understood, though some related works have been published in the literature [8,9,10,11,12,13,14]. Less attention has been paid to the crystallization of the component with a lower melting point (T_m_), particularly in a partially miscible crystalline/crystalline blend system. This rests on the fact that the crystallization of the low-T_m_ component in this special kind of blend is actually affected not only by the miscibility varying with the composition but also by the pre-existing crystals of the high-T_m_ component.

In this work, we chose poly(vinylidene fluoride) (PVDF) and poly(ε-caprolactone) (PCL) as the model system of partially miscible crystalline/crystalline polymer blends. From the measurements of the cloud point and melting point depression, the PVDF/PCL blend was found to be miscible when the content of PVDF was less than 30 wt % [15]. Since the difference in the melting points between PVDF (ca. 167 °C) and PCL (ca. 56 °C) is large, the crystallization of PVDF is always completed before PCL. So the sequential crystallization of PVDF and PCL corresponds to the phase transition from the fully amorphous to the amorphous/crystalline, and then to the crystalline/crystalline state. The phase separation structure of PVDF/PCL was studied by taking into account the influences of the blend composition. Moreover, much more attention has been directed to the crystallization and morphology of the low-T_m_ component PCL by considering the effect of the presence of the pre-existing crystals of the high-T_m_ component PVDF. Finally, the crystallization behavior of PCL on highly orientated PVDF film was studied by POM and TEM.

## 2. Experimental Section

### 2.1. Materials

PCL (*M*_n_ = 45,000) and PVDF (*M*_n_ = 275,000) were purchased from Sigma-Aldrich Company. The melting points were measured to be 167 °C for PVDF and 56 °C for PCL. Both of them were used without further purification.

Blends of PCL and PVDF were prepared by solvent casting using DMF as a mutual solvent (total concentration was 20 mg/mL). Thin films for POM and SEM observations were prepared by solution casting on glass slide. The obtained films were heat-treated at 190 °C and then cooled to predetermined isothermal crystallization temperature. To check the epitaxial behavior of PCL with PVDF, a double-layered sample was prepared by transferring a thin layer of PCL onto the highly oriented PVDF substrate. The double-layered sample was heated to 85 °C for 15 min to erase the thermal history of PCL and then cooled to 30 °C for isothermal crystallization. The highly oriented PVDF film was prepared through a melt-drawing technique introduced by Petermann and Gohil [16].

### 2.2. Measurements

A JEM GEOL-100CX electron microscope operated at 100 kV, a Field Emission Scanning Electron Microscope (Hitachi S-4800, Japan) operated at 2.0 kV and an OLYMPUS BH-2 optical microscope were used for TEM, SEM and optical microscopy observation, respectively. For SEM, the films were immersed for 24 h in chloroform at room temperature to etch away PCL. Etching was necessary because the lamellar bundles of PVDF were buried in PCL and almost invisible. After etching, the lamellar morphologies of PVDF emerged. Thermal analysis was measured by means of differential scanning calorimetry (DSC) using the DSC-2000 equipped with an Intracooler system.

## 3. Results and Discussion

**Figure 1:**
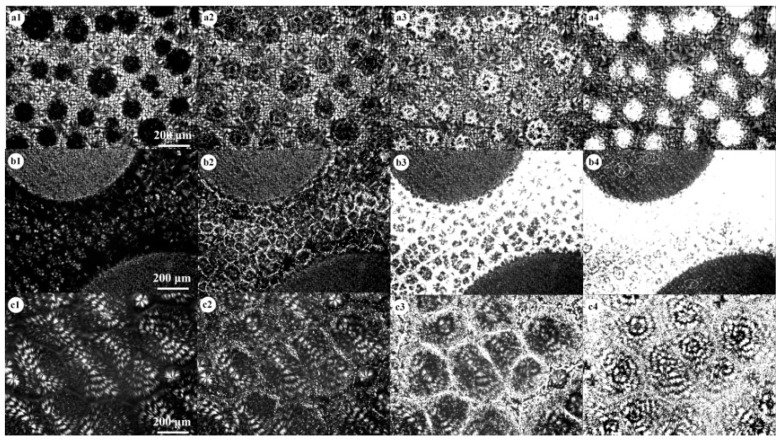
POM images of (**a1**–**a4**) 70/30, (**b1**–**b4**) 50/50 and (**c1**–**c4**) 30/70 PVDF/PCL blends. The blend samples were first cooled from 190 to 150 °C for the crystallization of PVDF and then quenched to 30 °C to crystallize PCL.

Figure 1 shows the blend composition effect on the phase separation and the crystallization of PVDF and PCL. Figure 1(a1,b1,c1) show the morphologies of 70/30, 50/50 and 30/70 PVDF/PCL blends at 150 °C, at which temperature the crystallization of PVDF has been completed but PCL is still in the melt state. In the 70/30 blend shown in Figure 1(a1), the PVDF-rich phase was continuous and the PCL-rich phase domains were finely dispersed within the PVDF-rich phase, indicating that the two components are immiscible in PVDF-rich compositions. In addition, the phase separation morphology indicated that the mechanism of nucleation and growth governs the development of phase separation of the 70/30 mixture (see Supplementary Materials). In the 50/50 blend, PVDF was still immiscible with PCL and the two components formed bicontinuous phases (see Figure 1(b1) and Supplementary Materials). In the 30/70 blend shown in Figure 1(c1), the PVDF component formed uniform spherulites with increased ring spacing and the melt of PCL was expelled into the interspherulitic, interfibrillar or interlamellar regions of the PVDF spherulites. The open spherulitic morphology of PVDF observed here can be often found in its miscible blend with other low-T_m_ crystallizable blends, suggesting that PVDF is miscible with PCL in PCL-rich compositions. These findings are in accordance with the report of Jo et al. [15]. However, it should be noticed that some open PVDF spherulites could be observed within the PCL-rich phase in the 70/30 and 50/50 blends, suggesting that the PCL-rich phase also contains small amounts of PVDF and the two components are miscible in the PCL-rich phase.

Figure 1(a2–a4,b2–b4,c2–c4) show the crystallization of PCL in the 70/30, 50/50 and 30/70 blends, respectively, when the samples were cooled to 50 °C. For the 70/30 blend, it was difficult to observe the crystallization of PCL in the PVDF-rich phase because of the low resolution of the optical microscopy. Therefore, the crystallization of PCL in the PCL-rich phase in the 70/30 blend has been paid greater attention. As shown in Figure 1(a2), the crystallization of PCL occurred in the interspherulitic region in the PCL-rich phase or the interfacial region between the PVDF-rich phase and the PCL-rich phase. Subsequently, the crystallization of PCL proceeded within the PVDF spherulites in the PCL-rich phase (see Figure 1(a3)). For the 50/50 blend, it was found that the crystallization of PCL still occurred in the interspherulitic region and then proceeded in the PCL-rich phase (see Figure 1(b2)). It was noticed that the PCL started to crystallize in the PVDF-rich phase after its complete crystallization in the PCL-rich phase (see the white circles in Figure 1(b4)). This indicated that the PCL was probably located in the interlamellar regions of the PVDF in the PVDF-rich phase and the crystallization of this part of the PCL was confined [17]. The crystallization of the PCL in the 30/70 blend was similar to that in the PCL-rich phase in the 70/30 and 50/50 blends. As seen in Figure 1 (c2–c4), the crystallization of the PCL occurred in the interspherulitic regions and then proceeded in the interfibrillar or interlamellar regions.

**Figure 2:**
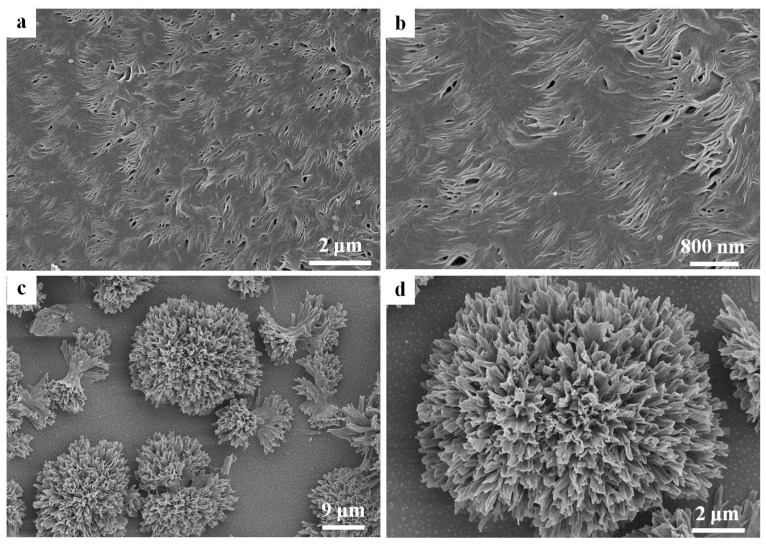
SEM images of (**a**,**b**) PVDF-rich phase and (**c**,**d**) PCL-rich phase in the 70/30 PVDF/PCL blend crystallized at 150 °C. The PCL component has been etched away.

For a better understanding of the morphological details of the PVDF/PCL blend systems, a further study of the internal structures of PVDF spherulites is necessary. Figure 2 shows the SEM micrographs of the spherulitic morphologies of the 70/30 blend melt-crystallized at 150 °C. Figure 2a,b shows the spherulitic morphologies of PVDF in the PVDF-rich phase. As seen in Figure 2a or Figure 2b, the PVDF spherulite is composed of the twisting lamellar bundles that are viewed alternately edge-on and face-on. In addition, the arrangement of the lamellae is very tight. Figure 2c,d show the spherulitic morphologies of PVDF in the PCL-rich phase. As seen in Figure 2c,d, the open PVDF spherulites are well dispersed in the PCL-rich phase. Some of PVDF spherulites grew into corals, indicating that a large amount of the PCL component was located in the intraspherulitic region. However, the other PVDF spherulites just grew into sheaves due to the lack of raw materials.

**Figure 3:**
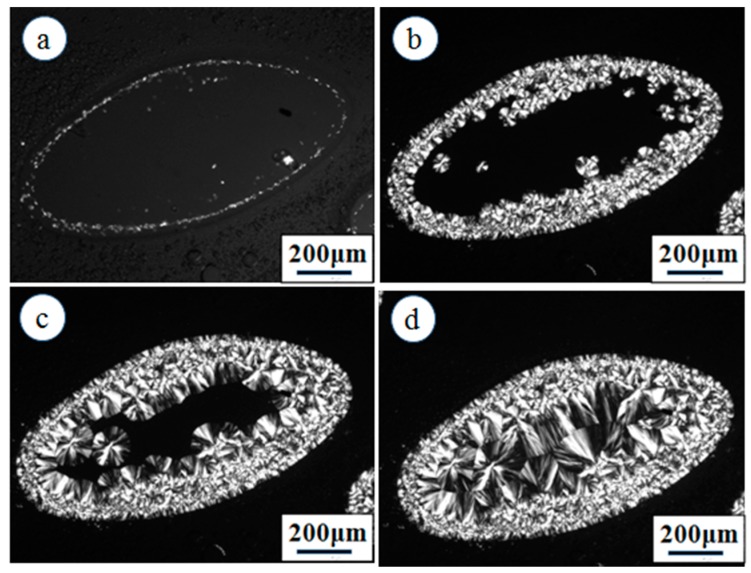
Crystallization of PCL at 30 °C for (**a**) 2 min, (**b**) 5 min, (**c**) 10 min and (**d**) 20 min after the crystallization of PVDF at 100 °C in PVDF/PCL (70/30) blend.

**Figure 4:**
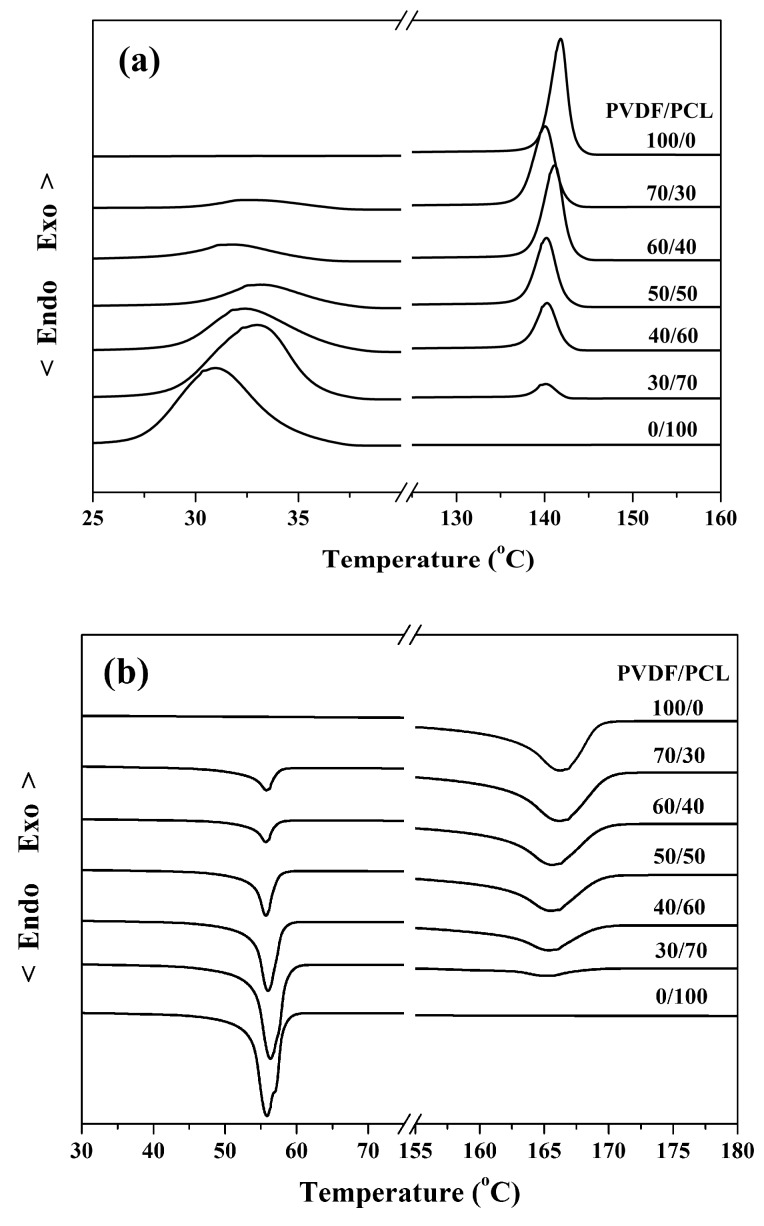
(**a**) DSC cooling traces of PVDF/PCL blends at 10 °C/min and (**b**) subsequent melting behavior at 10 °C/min.

Next, the effect of the pre-existing PVDF spherulites on the crystallization of PCL was further investigated. One possible scenario is that the crystallization rate of PCL will be accelerated due to the nucleation effect of PCL crystals induced by PVDF crystals. Another possible scenario is that the crystallization of PCL will be inhibited if the pre-existing PVDF crystals have no nucleation ability. Figure 3 shows the crystallization of PCL at 30 °C after the complete crystallization of PVDF at 100 °C in the 70/30 PVDF/PCL blend. The PCL-rich phase is located in the central region and surrounded by the PVDF-rich phase. As seen in Figure 3a,b, the crystallization of PCL occurs along the border between the PVDF-rich phase and PCL-rich phase. Because of the high nucleation density, a transcrystalline layer of PCL came into being. Subsequently, the transcrystalline layer kept growing in the PCL-rich phase until colliding with the PCL spherulites. The formation of the transcrystalline layer of PCL clearly indicates that the pre-existing PVDF crystals have a nucleation effect on the crystallization of PCL. Figure 4 shows DSC cooling traces of PVDF/PCL blends at 10 °C/min (a) and the subsequent melting behavior at 10 °C/min (b). As shown in Figure 4, both the crystallization and melting temperatures of the PVDF component in any of the PVDF/PCL blends were lower than neat PVDF, which suggests that some degree of miscibility exists for all compositions. In addition, the crystallization temperature of PCL in its blends with PVDF was higher than neat PCL, indicating that the pre-existing PVDF crystals promoted the crystallization of PCL.

**Figure 5:**
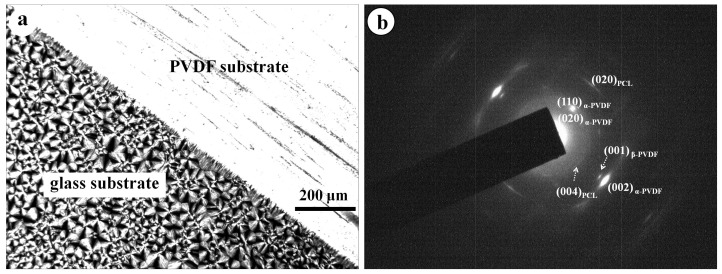
(**a**) Optical micrograph shows the morphologies of PCL melt-crystallized at 30 °C on glass slide (lower left corner) and PVDF substrate (upper right corner); (**b**) Electron diffraction pattern of a PCL/PVDF double-layered film.

The above POM and DSC results have clearly shown that in the PVDF/PCL blend, the pre-existing PVDF crystals acted as nucleating agents and could accelerate the crystallization of PCL. Next, the nucleation mechanism of PCL on PVDF crystals was investigated from the perspective of epitaxial crystallization. Figure 5a shows the different supermolecular structures of PCL crystallized on the glass and PVDF surfaces, respectively. On the glass substrate (in the lower-left corner of Figure 5a), PCL crystallized in a spherulitic structure. On the PVDF substrate (in the upper-right corner of Figure 5a), the PCL crystals showed much stronger birefringence with respect to the PCL spherulites formed on the glass substrate. In addition, no individual PCL crystals could be identified. This implies that the PVDF substrate exhibits strong nucleation ability toward PCL. It is documented that α-form PVDF crystals show the monoclinic unit cell with dimensions of a = 4.96 Å, b = 9.64 Å and c = 4.62 Å and PCL crystals show the orthorhombic unit cell with dimensions of a = 7.47 Å, b = 4.94 Å and c = 17.05 Å [18,19]. Figure 5b shows an electron diffraction pattern of a PCL/PVDF double layer. As seen in Figure 5b, the appearance of sharp and well-defined reflection spots of both PCL and PVDF on the electron diffraction pattern confirms that both PCL and PVDF substrate layers are highly oriented. In addition, the alignment of the [004] of PCL along the [002] direction of PVDF, as well as the [020] of PCL along the [020] direction of PVDF, reflects a parallel orientation between PCL and PVDF chains. The special parallel chain orientation of PCL and PVDF can be explained well in terms of a two-dimensional lattice matching. The lattice distances of the (004)_PCL_ and (001)_PVDF_ lattice planes are 4.32 and 4.62 Å, respectively. The mismatching between them is 6.4%. Furthermore, excellent matching can also be found between the lattice distances of the (010)_PCL_ and (020)_PVDF_ lattice planes with a mismatching of 3.2%.

## 4. Conclusions

The miscibility of the PVDF/PCL blend, the spherulitic morphology of the PVDF, and the crystallization behavior of the PCL in the PVDF/PCL blend as well as on the highly orientated PVDF substrate were studied by means of POM, DSC, SEM and TEM. It was found that the miscibility of the PVDF/PCL blend varies with the compositions. PVDF and PCL were immiscible in the PVDF-rich blend but they were miscible in the PCL-rich blend. In addition, the spherulitic morphology of PVDF was also affected by the blend ratio. In the PVDF-rich blend, PVDF formed tiny and compact spherulites in the PVDF-rich phase and crystallized in an open spherulitic structure in the PCL-rich phase. However, only open spherulites of PVDF were formed in the PCL-rich blend. The pre-existing PVDF crystals acted as the nucleation agent for PCL and promoted the subsequent crystallization of PCL. In the immiscible PVDF-rich blend, the crystallization of PCL occurred at the phase interface and then proceeded within the PCL-rich phase. In the miscible PCL-rich blend, PCL crystallized first in the interspherulitic region and then in the interfibrillar or interlamellar region. For all compositions, the nonisothermal crystallization temperature of the PCL component in the PVDF/PCL blend was higher than that of neat PCL at the same cooling rate. Finally, the strong nucleation ability of PVDF crystals toward PCL was further proved by the occurrence of heteroepitaxy and the transcrystallization of PCL on the PVDF substrate. The electron diffraction results show that there exists perfect two-dimensional lattice matching between PVDF and PCL crystals.

## Supplementary Materials

The following are available online at www.mdpi.com/2073-4360/9/2/42/s1, Figure S1: Phase separation morphologies in the 70/30 and 50/50 PVDF/PCL blend at 190 °C.

## References

[1] Si P., Luo F.L., Luo F.H. (2016). Miscibility, morphology and crystallization behavior of poly(butylene succinate-co-butylene adipate)/poly(vinyl phenol)/poly(l-lactic acid) blends. Polymers.

[2] Lorenzo M.L.D. (2003). Spherulite growth rates in binary polymer blends. Prog. Polym. Sci..

[3] Cho S.J., Cho J.H., Lee K.H. (2016). Phase behavior and its effects on crystallization in a poly(trimethylene terephthalate)/phenoxy resin blend. Polymers.

[4] Takala A., Takala P., Seppälä J., Levon K. (2015). Interdiffusion and spinodal decomposition in electrically conducting polymer blends. Polymers.

[5] Higgins J.S., Tambasco M., Lipson J.E.G. (2005). Polymer blends: Stretching what we can learn through the combination of experiment and theory. Prog. Polym. Sci..

[6] Lipatov Y.S. (2002). Polymer blends and interpenetrating polymer networks at the interface with solids. Prog. Polym. Sci..

[7] Ha C.S., Cho W.J. (2002). Miscibility, properties, and biodegradability of microbial polyester containing blends. Prog. Polym. Sci..

[8] Yang J.J., Pan P.J., Hua L., Feng X., Yue J.J., Ge Y.H., Inoue Y. (2012). Effects of crystallization temperature of poly(vinylidene fluoride) on crystal modification and phase transition of poly(butylene adipate) in their blends: A novel approach for polymorphic control. J. Phys. Chem. B.

[9] Yang J.J., Pan P.J., Hua L., Zhu B., Dong T., Inoue Y. (2010). Polymorphic Crystallization and phase transition of poly(butylene adipate) in its miscible crystalline/crystalline blend with poly(vinylidene fluoride). Macromolecules.

[10] Wang T.C., Li H.H., Wang F., Yan S.K., Schultz J.M. (2011). Confined growth of poly(butylene succinate) in its miscible blends with poly(vinylidene fluoride): Morphology and growth kinetics. J. Phys. Chem. B.

[11] Qiu Z.B., Yan C.Z., Lu J.M., Yang W.T., Ikehara T., Nishi T. (2007). Various crystalline morphology of poly(butylene succinate-co-butylene adipate) in its miscible blends with poly(vinylidene fluoride). J. Phys. Chem. B.

[12] Wang H.J., Gan Z.H., Schultz J.M., Yan S.K. (2008). A morphological study of poly(butylene succinate)/poly(butylene adipate) blends with different blend ratios and crystallization processes. Polymer.

[13] Wang H.J., Feng H.P., Wang X.C., Du Q.C., Yan C. (2015). Crystallization Kinetics and Morphology of Poly(vinylidene fluoride)/Poly(ethylene adipate) Blends. Chin. J. Polym. Sci..

[14] Madbouly S.A. (2007). Isothermal crystallization kinetics in binary miscible blend of poly(ε-caprolactone)/tetramethyl polycarbonate. J. Appl. Polym. Sci..

[15] Jo W.H., Park S.J., Kwon I.H. (1992). Phase behavior of poly(ε-caprolactone)/poly(vinylidene fluoride) blends. Polym. Int..

[16] Petermann J., Gohil R.M. (1979). A new method for the preparation of high modulus thermoplastic films. J. Mater. Sci..

[17] Kaito A., Iwakura Y., Hatakeyama K., Li Y.J. (2007). Organization of oriented lamellar structures in a miscible crystalline/crystalline polymer blend under uniaxial compression flow near the melting temperature. Macromolecules.

[18] Martins P., Lopes A.C., Lanceros-Mendez S. (2014). Electroactive phases of poly(vinylidene fluoride): Determination, processing and applications. Prog. Polym. Sci..

[19] Hasegawa R., Takahashi Y., Chatani Y., Tadokoro H. (1972). Crystal structures of three crystalline forms of poly(vinylidene fluoride). Polym. J..

