# Supplementary material for: Crystallization of Poly(ε-caprolactone) in Poly(vinylidene fluoride)/Poly(ε-caprolactone) Blend
Source: Polymers (Basel). 2017 Jan 28;9(2):42. doi: 10.3390/polym9020042 (PMC6432374; doi:10.3390/polym9020042)
Supplement: Supplementary file 1 [file polymers-09-00042-s001.pdf]

Supplementary Materials: Crystallization of Poly(ε-caprolactone) in Poly(vinylidene fluoride)/Poly(ε-caprolactone) Blend

Yang Kong, Yangmin Ma, Lele Lei, Xuechuan Wang and Haijun Wang

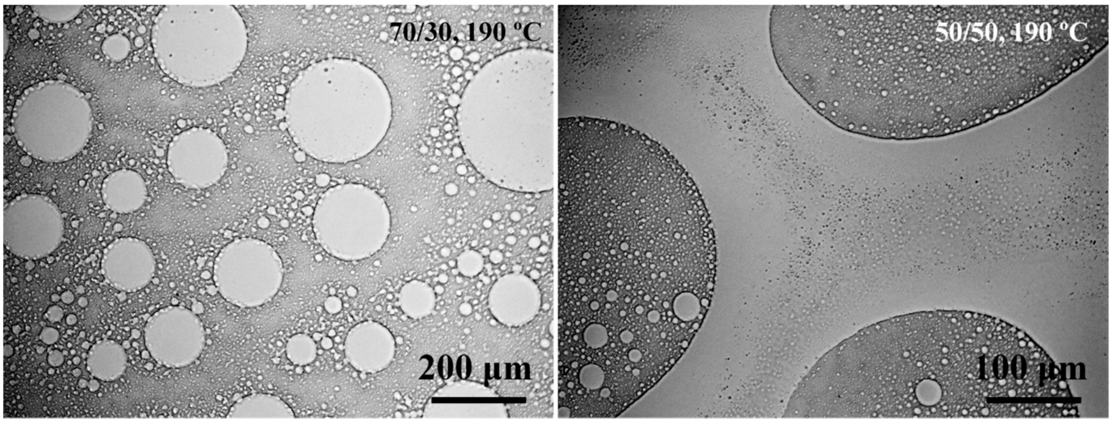

Figure S1. Phase separation morphologies in the 70/30 and 50/50 PVDF/PCL blend at 190 °C.
